# Neuropsychiatric profile in tuberous sclerosis complex patients with epilepsy

**DOI:** 10.3389/fped.2024.1436061

**Published:** 2025-01-15

**Authors:** Mahmoud Fawzi Osman, Faisal Hadid, Tawfeg ben Omran, Munira Aden, Fatima Al-Maadid, Sondos Altaraqji, Khalid Mohamed, Ruba Benini

**Affiliations:** ^1^ Division of Pediatric Neurology, Sidra Medicine, Doha, Qatar; ^2^ Neurology Division, Pediatric Department, Weil-Cornell Medical College Qatar, Doha, Qatar; ^3^ Hamad Medical Corporation, Sidra Medicine, Doha, Qatar

**Keywords:** tuberous sclerosis complex (TSC), epilepsy, intellectual disability, intellectual, autism, neuropsychiatric disorders

## Abstract

**Background:**

Tuberous sclerosis complex (TSC) is an autosomal dominant disorder characterized by mutations in the *TSC1* or *TSC2* genes, leading to dysregulation of the mTOR pathway and multisystemic manifestations. Epilepsy is a common neurologic feature of TSC, frequently accompanied by neuropsychiatric comorbidities. Understanding the relationship between epilepsy severity, TSC-associated neuropsychiatric disorders (TAND), and cognitive outcomes is crucial for optimizing patient care.

**Methods:**

A retrospective study was conducted at a pediatric tertiary care hospital in Qatar, involving 38 TSC patients (20 female, 18 male) aged 1–18 years, diagnosed between October 2018 and March 2020. Epilepsy severity was assessed using the Early Childhood Epilepsy Severity Scale (E-Chess), and TAND was evaluated using the TAND checklist. Genetic analysis was performed for all patients, and statistical analyses were used to explore correlations between epilepsy severity, TAND, and cognitive outcomes.

**Results:**

The majority (82%) of TSC patients had epilepsy, with a mean onset age of 9.2 months. Uncontrolled seizures were associated with higher rates of intellectual disability and more pronounced TAND manifestations compared to controlled seizures. Autism spectrum disorder (ASD) was reported in 42% of the cohort, with significant correlations found between epilepsy severity and ASD-related domains on the TAND checklist. Intellectual disability was prevalent (67.6%), with variability attributed to genetic background and early severe neurological presentations.

**Discussion:**

This study reinforces the link between epilepsy severity and neuropsychiatric comorbidities in TSC, confirming earlier findings. Significant correlations were observed between epilepsy severity and ASD-related domains, and the high prevalence of intellectual disability in TSC patients was highlighted. However, the relationship between ASD, TSC, and epilepsy remains complex and requires further investigation. Despite advances in treatment options, including mTOR inhibitors and newer antiepileptic drugs, unmet needs remain in the comprehensive care of TSC patients. Optimizing seizure control is a clear priority, but equally important is the need for addressing the cognitive and behavioral components of TAND. Early intervention with tailored, multidisciplinary approaches including neurology, psychiatry, psychology, and educational specialists could mitigate the long-term impact of these comorbidities, particularly in children. These approaches must be individualized to each patient's unique set of challenges, emphasizing not only seizure control but also psychosocial support and educational adaptation to improve their overall quality of life.

**Conclusion:**

This study sheds light on the intricate interactions between epilepsy severity, neuropsychiatric manifestations, and cognitive outcomes in TSC patients. The findings emphasize the need for tailored management approaches, focusing on early seizure control and comprehensive multidisciplinary care. Further research is required to clarify the mechanisms underlying these associations and to develop targeted interventions for improving the quality of life for individuals with TSC and epilepsy.

## Introduction

Tuberous sclerosis complex (TSC) is an autosomal dominant disorder caused by mutations in the *TSC1* (hamartin) or *TSC2* (tuberin) genes, which regulate the mTOR pathway, crucial for cellular growth and proliferation (1–5). As a multisystemic condition, TSC affects various organs, including the brain, heart, skin, kidneys, lungs, and eyes (6). Cardiac and neurological manifestations are often the earliest signs, with cardiac rhabdomyomas sometimes detected in fetal life (7). Neurologic and psychiatric symptoms, particularly seizures, are common and contribute significantly to morbidity in TSC patients (6, 8–10). More than 75% of individuals with TSC experience epilepsy, which often precedes neuropsychiatric conditions such as intellectual disability and autism spectrum disorder (ASD) (11, 12). The severity of epilepsy has been strongly linked to the development of these neuropsychiatric comorbidities, including cognitive deficits and behavioral disturbances (13–16). Neuroimaging in TSC frequently reveals cortical tubers malformations characterized by disorganized cortical architecture and dysplastic neurons, which are closely associated with epilepsy and neuropsychiatric outcomes (17). The number and distribution of these tubers correlate with the severity of neuropsychiatric symptoms (17). These co-morbidities increase the overall burden of illness in TSC patients, and translate to an overall negative impact on quality of life (QOL) for both patients and their families.

In this study, the Early Childhood Epilepsy Severity Scale (E-Chess) and the TSC-associated neuropsychiatric disorder (TAND) checklist were used to quantify epilepsy severity and neuropsychiatric manifestations respectively in a cohort of 31 patients with TSC and epilepsy in Qatar. E- Chess is a standardized scale that was developed to quantify the severity of epilepsy in children participating in the Tuberous Sclerosis 2000 Study, a longitudinal study of cases of tuberous sclerosis (TS) diagnosed in the United Kingdom between January 2001 and December 2005 (13). The TAND Check list has been shown to be an effective and accurate clinical tool for TAND screening and was developed in a retrospective study at Pediatric Neurology Unit of an Italian Tertiary Care Hospital (13).

Although there is extensive literature on the clinical and genotypic characterization of TSC, there remains many research gaps pertaining to the rare symptoms and the co-morbidities inherent of this disease and the complex interactions between them. This includes a need for clarifying the relationship between epilepsy and neuropsychiatric manifestations, the two most common co-morbidities, and the risk factors that modulates their expression. This present study aims at addressing this latter gap.

## Methods

### Study population

This retrospective population-based study was conducted between October 2018 and March 2022 at Sidra Medicine, the sole pediatric tertiary care hospital for children in Qatar. Sidra Medicine is the primary referral center for children with complex neurological disorders in Qatar, where all pediatric patients with TSC are followed. The study focused on individuals diagnosed with tuberous sclerosis complex (TSC), ranging in age from one year to 18 years. The study protocol received approval from the Institutional Review Board (IRB).

A total of 38 pediatric patients (18 males and 20 females) who were assessed and diagnosed with TSC at the TSC clinic were initially included in the study. The diagnosis of TSC was made according to the International Tuberous Sclerosis Consensus criteria. Among these patients, 31 individuals with a confirmed diagnosis of epilepsy were included in the study, while those without epilepsy were excluded as the E-Chess scale, used in correlation with TAND, cannot be applied to patients without epilepsy.

To assess the neuropsychiatric manifestations associated with TSC, the TSC-associated neuropsychiatric disorder (TAND) checklist was employed. The TAND checklist, developed by the Neuropsychiatry Panel at the 2012 Tuberous Sclerosis Complex International Consensus Conference and reported by de Vries et al., covers various domains, including behavioral, intellectual, neuropsychological, psychiatric, academic, and psychosocial aspects. Responses to the TAND checklist were recorded by a specialized clinical nurse through interviews with the families. This recording took place either during the child's follow-up appointments at the hospital or through telephone interviews at mutually convenient times.

The severity of epilepsy was quantified using the Early Childhood Epilepsy Severity Scale (E-Chess), which was completed by a neurologist based on a thorough review of patient charts.

In summary, this study focused on a cohort of TSC patients with epilepsy, utilizing the TAND checklist to assess neuropsychiatric manifestations and the E-Chess scale to quantify epilepsy severity. The study was conducted at Sidra Medicine, where comprehensive evaluations and follow-up care for TSC patients are provided.

### Genetic analysis

Genetic tests were conducted for all 38 patients at a certified College of American Pathologists (CAP) laboratory located abroad. The genetic results were obtained from the patients’ medical charts, ensuring access to accurate and reliable genetic information for analysis.

### Epilepsy analysis

To evaluate the severity of epilepsy at the last follow-up, the Early Childhood Epilepsy Severity Scale (E-Chess) was utilized. The E-Chess scoring parameters included:

• Age at epilepsy onset (<1 year, 1–3 years, >3 years)
• Type of epilepsy (focal or generalized seizures, epileptic encephalopathy)
• Frequency of seizures in the last 6 months (no seizures, monthly, weekly, >1 per day)
• Number of antiepileptic drugs taken at the last follow-up (0, 1–2, 3, >3)
• Response to treatment (seizure-free with or without antiepileptic drugs, drug-resistant)

Comparisons were made between the characteristics of the overall epilepsy cohort and those with epilepsy diagnosed before 2 years of age (early onset seizure group). Based on the E-Chess score, patients were classified into two groups: favorable outcome (total score: 2–7) and unfavorable outcome (total score: 8–15), indicating the severity of epilepsy.

### Statistical analysis

The data was coded and entered into SPSS 22.0 software (SPSS Inc., Chicago, IL, USA). The patients who had TSC and an unfavorable outcome on the E-Chess were considered case patients, while those with TSC and a favorable outcome on the E-Chess were considered control patients. Categorical variables were analyzed using the Chi-squared (*χ*^2^) test. All reported *p*-values were based on two-tailed tests of significance, with *α* set at 0.05, indicating the level of statistical significance.

## Results

The results of our study provide a comprehensive understanding of the relationship between Tuberous Sclerosis Complex (TSC), epilepsy, and neuropsychiatric manifestations. Our findings highlight the following key insights:

### High prevalence of epilepsy

Among the TSC patients included in the study, 31 (82%) were diagnosed with epilepsy. Demographic summery of the patients for gender; nationality and genetic workout (Table 1).

#### Diverse seizure types and onset

The onset of seizures ranged between 1 month and 4 years, with a mean of 9.5 months. Out of the 31 patients with epilepsy, 7 (18%) reported infantile spasms, while the remaining presented with pure focal seizures (11/31), both focal and generalized seizures (7/31) and pure generalized seizure (15/31) most commonly infantile spasms (11/15).

#### Early onset epilepsy

Early onset epilepsy, defined as the onset of seizures before the age of 2 years, occurred in 27/31 patients. The mean age of epilepsy presentation was 6.8 months for patients with infantile spasms, 9.5 months for focal epilepsy, and 12.8 months for generalized epilepsy.

#### Challenges of drug-resistant epilepsy

A majority of the patients, 23/31 (74.2%), exhibited drug-resistant epilepsy, which is characterized by persistent seizures despite being on two appropriate antiepileptic medications. Out of these patients, 7 underwent epilepsy surgery, and no patients were placed on the ketogenic diet to assist with seizure control. Controlled seizures were reported in only 8 (21%) patients. Drug-resistant epilepsy was more prevalent in patients with infantile spasms (11/11, 100%) compared to those with focal epilepsy (7/16, 40%).

### Comorbidity of autism spectrum disorders and attention deficit hyperactivity disorder

TSC patients exhibited a range of psychiatric manifestations and were typically diagnosed at a relatively young age, with most cases being identified before the age of 10 years. These manifestations included speech and language disorders, autism spectrum disorder (ASD), behavioral disorders, attention-deficit/hyperactivity disorder (ADHD), and various learning disabilities. Out of the 31 patients included in the study, 24/31 (77%) patients reported learning disabilities overall. Specifically, there was a prevalence of learning disabilities in reading, writing, spelling, mathematics, and memory, ranging from (8/24 patients) 33.3% in the favorable outcome group compared to (16/24 patients) 67% in the unfavorable outcome group (*p*-values <0.05).

ADHD was reported by 15 out of 31 (47%) patients with TSC and epilepsy. Furthermore, autism was present in 13 out of 31 (42%) patients with epilepsy, and it was more common among those with E-Chess scores >7. Domains related to ASD, such as poor eye contact, repetitive behaviors, absent or delayed communication, and difficulty getting along with peers, were also more prevalent in this group and showed statistical significance.

### Correlation between epilepsy severity and neuropsychiatric symptoms

Notably, the study established a significant correlation between the severity of epilepsy, as measured by the Early Childhood Epilepsy Severity Scale (E-Chess), and the presence of neuropsychiatric symptoms. Patients with more severe epilepsy were more likely to exhibit psychological manifestations and ASD-related symptoms, as indicated by the TSC-associated neuropsychiatric disorder (TAND) checklist (Table 2).

**Table 1:** Demographics and clinical information on patient cohort.

Summary of patient demographics and clinical features	
Gender	
Male	47% (18/38)
Female	53% (20/38)
Nationality	
** **Qatari	21% (8/38)
** **Non-Qatari	79% (30/38)
Mean age at diagnosis	4 months
Genotype	
TSC 1	2.6% (1/38)
TSC 2	86% (33/38)
Negative genetic testing	10.5% (4/38)

**Table 2:** Comparison of tAND manifestations between favorable and unfavorable epilepsy outcome groups.

TAND checklist categories	Favorable epilepsy outcome (E-chess 2–7)	Unfavorable epilepsy outcome (E-chess 8–15)	*P*-value
Temper tantrum	0/31	5/31 (16%)	0.0197^a^
Aggressive outbursts	0/31	8/31 (25.8%)	0.00244^a^
Anxiety	0/31	1/31 (3.2%)	0.3134
Depressed mood	0/31	0/31	NA
Self-injury	0/31	1/31 (3.2%)	0.3134
Difficulties paying attention	5/31 (16%)	14/31 (45%)	0.01316^a^
Overactivity/hyperactivity	1/31 (3.2%)	10/31 (32%)	0.002772^a^
Impulsivity	2/31 (6%)	6/31 (19%)	0.1297
Absent or delayed onset of language to communicate	5/31 (16%)	15/31 (48%)	0.006592^a^
Poor eye contact	2/31 (6%)	10/31 (32%)	0.01012^a^
Repetitive behaviors	1/31 (3.2%)	2/31 (6%)	0.554
Sleep difficulties	0/31	5/31 (16%)	0.0197^a^
Difficulties with eating	0/31	5/31 (16%)	0.0197^a^
Extreme shyness	2/31 (6%)	0/31	0.1506
Mood swings	1/31 (3%)	9/31 (29%)	0.005738^a^
Repeating words or phrases over and over again	0/31	1/31 (3%)	0.3134
Difficulties getting on with peers	1/31 (3%)	10/31 (32%)	0.002772^a^
Very rigid or inflexible about how to do things	1/31 (3%)	6/31 (19%)	0.0448^a^
Restlessness or fidgetiness	0/31	1/31 (3%)	0.3134
ASD	3/31 (9.6%)	10/31 (32%)	0.02897^a^

^a^ Denotes clinical significance *p* < 0.05.

### Limited reporting of anxiety and depressed mood

Interestingly, anxiety and depressed mood were less commonly reported among TSC patients in our cohort (1 out of 31). This observation could be influenced by various factors, such as the age of the patients and cultural factors affecting symptom reporting.

## Discussion

This study highlights the intricate relationship between psychiatric manifestations, cognitive impairments, and epilepsy severity in individuals with Tuberous Sclerosis Complex (TSC) and epilepsy. The findings shed light on several critical aspects.

Our patient cohort showed that TSC 2 was the most common etiology (86%); this comparable to reported prevalence (2) (∼51%–82%). Generally, it is reported that patients with *TSC2* mutations tend to have more severe disease than those with *TSC1* mutations (2); this can explain the severity of disease presentations in our patients. 31 patients (82%) were diagnosed with epilepsy; this is also comparable to other studies (2, 6–8) showing the prevalence of epilepsy to range from 62% to 93%. Similar to other studies the onset of epilepsy in our cohort was in the first 4 years of life (7, 8). Notably however, only 18% of our patients presented with infantile spasms, which is lower than other studies (6–8) (37%–50%). Consistent with previous studies, statistically significant results were found on epilepsy-TAND neuropsychiatric correlations, both on E-chess score (*p* *<* *0.05*) and the unfavorable epilepsy outcome, which was consistent to the previous studies (8, 12).

### Learning disabilities

77% of patients with epilepsy have learning disabilities, particularly in domains related to reading, writing, spelling, and mathematics. Learning disabilities was more prevalent (*p* < 0.05) in TSC patients with unfavorable outcome compared to favorable outcome group similar to other studies (6–8, 18–21). The association between learning disabilities and epilepsy severity underlines the intricate interplay between cognitive functioning and epilepsy in TSC patients.

### Autism Spectrum disorder (ASD)

In this study, there was a higher prevalence of ASD in TSC patients with epilepsy 13/31 (42%) and ASD was more prevalent in patients with unfavorable epilepsy 10/13 (77%) compared to 3/13 (23%) with favorable outcome as indicated by higher E-Chess scores (*p* = 0.02897). This finding strengthens the link between epilepsy severity and the presence of neurodevelopmental disorders such as ASD, along with associated ASD-related domains. Similar result reported before (12, 22).

### Anxiety and depressed mood

In our study, limited reports of anxiety and depressed mood were observed among the cohort. This may suggest that these symptoms are less prominent in this specific patient population. One potential explanation could be the higher socioeconomic status in Qatar, which provides better access to healthcare, mental health support, and comprehensive care for TSC patients. Additionally, the strong familial and social support systems common in this region might play a protective role against anxiety and mood disorders. Cultural factors, including societal perceptions of mental health, may also lead to underreporting of these symptoms by patients or caregivers (18). Lastly, methodological limitations, such as reliance on caregiver reports and the possible lack of in-depth psychiatric evaluations, may have contributed to the lower detection of anxiety and depression in this cohort. A more detailed, culturally sensitive assessment of mental health in TSC patients could offer a clearer understanding of the prevalence and impact of these symptoms.

### Challenges in epilepsy management

The study's identification of drug-resistant epilepsy in a significant proportion of patients highlights the complex challenges in managing epilepsy within the context of TSC. The utilization of alternative treatments like epilepsy surgery and the ketogenic diet underscores the need for diverse approaches to address refractory seizures. This is also reported before in another studies (8–15).

### Multidisciplinary care

The findings underscore the importance of comprehensive multidisciplinary care for individuals with TSC and epilepsy. Early identification and management of psychiatric symptoms, cognitive impairments, and neurodevelopmental disorders are crucial for optimizing the overall well-being and long-term outcomes of these patients.

### Implications for future research

The study's insights open avenues for further research into the underlying mechanisms linking epilepsy severity, neuropsychiatric manifestations, and cognitive impairments in TSC patients. Future studies could delve deeper into understanding the neurobiological pathways and interactions that contribute to these comorbidities, and potential unveil therapeutic targets to address both epilepsy and neuropsychiatric manifestations of TSC.

Screening for epilepsy before the onset of clinical seizures in infants with tuberous sclerosis complex (TSC) is a topic of ongoing debate. Some studies suggest that early detection through EEG monitoring and other diagnostic tools enables timely intervention, potentially reducing the risk of developmental delays and cognitive impairment (19, 23, 24). These studies indicate that by diagnosing and managing TSC early, healthcare providers can implement personalized treatment plans that support optimal growth and development, thereby improving the quality of life for these young patients (25).

However, there is insufficient evidence to conclusively support the effectiveness of early EEG screening and treatment in improving long-term developmental outcomes in children with TSC. Not all subclinical seizures detected by EEG necessitate intervention, as some infants may not develop clinically significant epilepsy (25). Additionally, concerns exist regarding the potential risks associated with early or overtreatment, such as medication side effects and the psychological impact of diagnosing a child with a chronic condition at a very young age (21). Further studies are needed to clarify the benefits and risks of early EEG screening and intervention to guide more effective and personalized management strategies for children with TSC.

### Limitation of the study

#### Study limitations

This study has several limitations that should be acknowledged. First, the relatively small sample size, limited to 31 patients with epilepsy, may affect the generalizability of the findings to the broader TSC population. Second, the study relied on the TAND checklist and caregiver reports for the evaluation of neuropsychiatric symptoms, which may have introduced subjectivity and potential underreporting, particularly regarding mood disorders such as anxiety and depression. Third, the lack of detailed psychiatric evaluations or standardized mental health screening tools may have limited the accuracy of identifying more subtle or undiagnosed mental health issues. Finally, this study was conducted in a high-income setting with better access to healthcare services, which may not reflect the experiences of TSC patients in regions with fewer resources or less comprehensive care.

## In conclusion

Our study sheds light on the intricate relationships between epilepsy, neuropsychiatric manifestations, and TSC. The findings underline the multifaceted challenges faced by TSC patients and healthcare providers in managing epilepsy and its associated comorbidities. These results emphasize the need for tailored interventions that address both epilepsy control and the broader spectrum of neuropsychiatric symptoms, with the ultimate goal of improving the quality of life for individuals with TSC. By recognizing the intricate connections between epilepsy severity, cognitive functioning, and psychiatric manifestations, healthcare providers can offer more effective and holistic approaches to improve the lives of individuals with TSC and epilepsy.

## Data Availability

The original contributions presented in the study are included in the article/Supplementary Material, further inquiries can be directed to the corresponding author.
